# Tenofovir disoproxil fumarate-associated renal tubular dysfunction: noninvasive assessment of mitochondrial injury

**DOI:** 10.1097/QAD.0000000000001466

**Published:** 2017-05-11

**Authors:** Ryan Samuels, Carla Roca Bayerri, John A. Sayer, D. Ashley Price, Brendan A.I. Payne

**Affiliations:** a Wellcome Trust Centre for Mitochondrial Research; b Institute of Genetic Medicine, Newcastle University; c Department of Infection and Tropical Medicine, Newcastle upon Tyne Hospitals NHS Foundation Trust, Newcastle upon Tyne, UK.

**Keywords:** antiretroviral therapy, biomarkers, DNA, Fanconi syndrome, HIV, mitochondrial, renal insufficiency, tenofovir

## Abstract

**Objective:**

To determine whether tenofovir disoproxil fumarate (TDF)-associated renal tubular dysfunction is associated with evidence of mitochondrial injury in urine.

**Design:**

Single-centre cross-sectional observational study of HIV-positive outpatients.

**Methods:**

Biochemistry was performed on paired serum and urine samples. Mitochondrial DNA (mtDNA) was studied by real-time PCR and long-range PCR on cellular fractions of urine.

**Results:**

In total, 48 study participants were enrolled of whom half were TDF treated. Mean age was 43 years. 58% had estimated glomerular filtration rate at least 90, with no differences between ART treatment groups. Urinary phosphate wasting was common and independently associated with TDF exposure (*P* = 0.02). No study participants had low molecular weight proteinuria. Cellular mtDNA content in urine was heavily influenced by the cellularity of the sample. The mtDNA ‘common deletion’ mutation was detectable significantly more commonly in the urine of TDF exposed study participants compared with unexposed (13/22 TDF^+^ study participants (59%), 4/21 TDF^−^ (19%), *P* = 0.01). Common deletion levels were not associated with age, estimated glomerular filtration rate, or urinary phosphate wasting. No mtDNA measures were associated with current or nadir CD4^+^ lymphocyte counts, duration of disease or antiretroviral therapy, or historical exposure to nucleoside analogue reverse transcriptase inhibitors with systemic mitochondrial toxicity.

**Conclusion:**

The presence of mtDNA mutations in the context of TDF exposure adds weight to the hypothesis that TDF-associated renal damage is at least in part mitochondrially mediated. The assessment of mtDNA markers in urine may be a feasible noninvasive investigation for TDF-treated patients.

## Introduction

Tenofovir disoproxil fumarate (TDF) is a first-line antiretroviral drug globally. Generally, it is well tolerated; however, a minority of patients develop renal complications. These can manifest either as deterioration of estimated glomerular filtration rate (eGFR) or as proximal tubulopathy leading to renal Fanconi syndrome [1,2]. TDF-associated renal tubular pathology is thought to arise from the fact that the active metabolite of tenofovir becomes concentrated within the cells of the proximal tubule. Thus toxicity that does not manifest systemically may nevertheless affect proximal tubular function. Many of the older nucleoside analogue reverse transcriptase inhibitor (NRTI) antiretroviral drugs caused systemic mitochondrial toxicity through inhibition of the sole mitochondrial DNA (mtDNA) polymerase, pol γ, leading to mtDNA depletion (for example, zidovudine, stavudine, didanosine, zalcitabine) [3,4]. Subsequent work from our group and others has shown that these drugs may also cause mtDNA mutations. One of the commonest mtDNA mutations is known as the ‘common deletion’. This is a 4977 bp large-scale deletion in the major arc of the mitochondrial genome, occurring between two homologous 13 bp repeat sequences. Sporadic occurrences of the common deletion in the germline lead to Kearns-Sayre syndrome, but the common deletion has also been detected as a common somatic mutation which accumulates during ageing in a variety of tissues [5–8]. The common deletion has also been detected in the context of NRTI therapy [9].

Previous studies have suggested that TDF does not cause mtDNA depletion in standard cell lines *in vitro* or in preclinical animal testing [10,11]. However, studies of rodents exposed to TDF have shown mtDNA depletion that is specific to the kidney [12,13], and a human study of patients undergoing renal biopsy for TDF-induced renal toxicity showed ultrastructural mitochondrial abnormalities on electron microscopy [14]. Although renal biopsy is the gold standard for defining pathology, it is not feasible for widespread clinical use in patients with mild or subclinical toxicity. Recent work in inherited mtDNA defects has shown that they can often be detected in the urine of affected patients [15].

We, therefore, hypothesised that evidence of mitochondrial damage would be detectable in the urine of TDF-treated HIV-infected patients. If true, this would confirm that TDF can cause renal-specific mitochondrial toxicity, and provide a putative noninvasive marker for the clinical investigation of TDF-treated patients.

## Methods

All study participants gave written informed consent for participation in the study. The study was approved by the Research Ethics Committee. This was a single-centre cross-sectional study of HIV-infected adults attending for outpatient HIV care. Consecutive study participants were enrolled without selection for the presence or absence or renal toxicity. Study participants with severe impairment of renal function [(eGFR, CKD-EPI, chronic kidney disease epidemiology collaboration) < 30] were excluded. Demographic, clinical, and treatment data were obtained by case note review.

Paired serum and urine samples (random/unfasted) were collected for renal biochemistry. Phosphate wasting was expressed as tubular maximum resorption of phosphate/GFR (TmP to GFR; lower limit of normal 0.8–1.0 mmol/l depending on age and sex) [16]. Urate wasting was calculated as fractional excretion of urate (the percentage of urine to plasma urate normalized to creatinine; upper limit of normal 10%). Tubular proteinuria was assessed by urinary retinol-binding protein (normal range 0–15 mg/l).

An additional urine sample (40 ml) was collected for mtDNA analyses. This was centrifuged to pellet the cellular content and total nucleic acid extraction was performed from 200 μl of pellet (NucliSENS easyMag, bioMerieux, Marcy l’Etoile, France) into a 55 μl elution volume.

Real-time PCR (qPCR) was performed on a CFX platform (Bio-Rad, Hercules, California, USA). A target in *MT-ND1* was used to quantify mtDNA content (primers/probe: 5′-ACGCCATAAAACTCTTCACCAAAG-3′, 5′-GGGTTCATAGTAGAAGAGCGATGG-3′, 5′-HEX-ACCCGCCACATCTACCATCACCCTC-BHQ1–3′). The 4977 bp mtDNA ‘common deletion’ mutation was detected by an allele-specific qPCR assay (primers/probe: 5′-CCCACCATAATTACCCCCATAC-3′, 5′-GGAGTAGAAACCTGTGAGGAAAGG-3′, 5′-Cy5-CCTACCTCCCTCACCATTGG-BHQ2–3′). Cell content was quantified by a target in *b2M* (primers/probe: 5′-CACTGAAAAAGATGAGTATGCC-3′, 5′-AACATTCCCTGACAATCCC-3′, 5′-FAM-CCGTGTGAACCATGTGACTTTGTC-BHQ1–3′). qPCR was performed in 20 μl reaction volumes comprising 1 × iQ Multiplex Powermix (Bio-Rad), 300 nmol/l common deletion and *b2M* primers, 75 nmol/l *MT-ND1* primers, 200 nmol/l probes, and 2 μl DNA. Cycling conditions were: 95°C for 3 min, followed by 40 cycles of 95°C for 10 s, and 62.5°C for 1 min. qPCR reactions were performed in triplicate.

Long-range PCR was performed on a Veriti thermocycler (Applied Biosystems, Foster City, California, USA) in a 25 μl reaction comprising: 1 × PrimeSTAR GXL DNA buffer (Takara, Clontech Mountain View, California, USA), 200 μmol/l dNTP mix, 200 nmol/l primers and 1.25 U GXL polymerase (Takara, Clontech) with 1–5 μl DNA. Primers were: 5′-CCCTCTCTCCTACTCCTG-3′, 5′-CAGGTGGTCAAGTATTTATGG-3′. The reaction generates a ∼10 kb product in the presence of undeleted (full-length) mtDNA.

## Results

### Study participant characteristics and renal biochemistry

We enrolled 48 study participants. Mean age was 43 years and 35 (73%) were male. Twenty-four study participants (50%) were receiving TDF-based antiretroviral therapy (ART). Of the 24 study participants not receiving TDF, 11 were on other ART and 13 were ART naive. Study participants characteristics are shown in Table 1. Among study participants taking TDF, 12 (50%) were on boosting agents (11 ritonavir, one cobicistat). In total, 20 study participants had been exposed to one or more pol γ inhibiting NRTIs at some point in their treatment history. eGFR did not differ between groups. Measures of proximal renal tubular function were also similar with the exception of urinary phosphate wasting. Mean TmP/GFR (± SD) was significantly lower in TDF-treated study participants compared with unexposed (TDF^+^ 0.82 ± 0.28, TDF^−^ 1.02 ± 0.26, *P* = 0.02). Low TmP/GFR correlated strongly with serum hypophosphataemia (*r* 0.78, *P* < 0.001) and all study participants with hypophosphataemia had low TmP/GFR, implying that urinary phosphate wasting was a major driver of hypophosphataemia. The majority of study participants with evidence of both urinary phosphate and urate wasting (low TmP/GFR and high fractional excretion of urate) were TDF treated (TDF^+^ 8/22, 36%; TDF^−^ 2/21, 10%; *P* = 0.04).

### mtDNA analyses

**Fig. 1:**
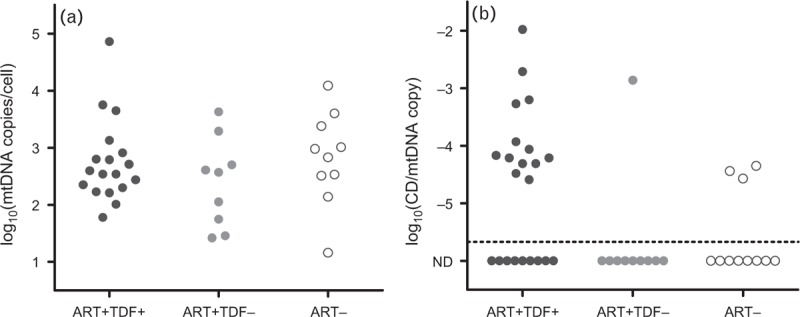
(a) mtDNA content in urine. (b) mtDNA common deletion mutation in urine. ART, antiretroviral therapy; ART^+^TDF^+^, TDF treated; ART^+^TDF^−^, treated with non-TDF therapy; ART^−^, antiretroviral naive; CD, common deletion; mtDNA, mitochondrial DNA; TDF, tenofovir disoproxil fumarate.

In total, 43 study participants had analysable mtDNA data. Urine is a highly heterogeneous tissue which may vary widely in cellular composition between individuals. As such, we observed no difference in urine mtDNA depletion between ART groups (log_10_ mtDNA copies/cell ± SD, TDF^+^ 2.75 ± 0.73, TDF^−^ 2.61 ± 0.81, *P* = 0.6, Fig. 1a). Full details of mtDNA depletion analyses are shown in supplementary online material.

Dotted line at −5.7 log_10_ copies/mtDNA corresponds to lower limit of assay. ND, not detected. Note: On each plot, dots represent individual study participants.

The mtDNA ‘common deletion’ was detectable in 17/43 urine samples (40%). Among cases with common deletion detected mean level of the common deletion mutation (± SD) was −3.86 ± 0.77 log_10_ common deletion per mtDNA genome copy. Presence of common deletion was significantly associated with TDF exposure (common deletion detected in 13/22 TDF-treated study participants (59%) compared with 4/21 TDF unexposed (19%, *P* = 0.01) Fig. 1b). Among TDF-treated study participants, mean duration of TDF exposure was significantly longer in those study participants with detectable common deletion (common deletion^+^ 63 ± 29 months, common deletion^−^ 32 ± 29, *P* = 0.02). Of note, detectability of common deletion was not confounded by the cellular content of urine. Presence of common deletion did not vary with past exposure to NRTIs with systemic mitochondrial toxicity [8/19 exposed study participants showed common deletion detectable (42%), compared with 9/24 unexposed study participants (38%)]. There was no association with study participant age (common deletion^+^ study participants 46.2 ± 11.0 years, common deletion^−^ 42.5 ± 13.1 years, *P* = 0.3), current CD4^+^ lymphocyte count (common deletion^+^ 577 ± 297 cells/μl, common deletion^−^ 570 ± 308 cells/μl, *P* = 0.9), eGFR (common deletion^+^ 93 ± 17, common deletion^−^ 96 ± 21, *P* = 0.8), or TmP/GFR (common deletion^+^ 0.90 ± 0.31, common deletion^−^ 0.96 ± 0.29, *P* = 0.5). There were trends toward associations between the presence of the common deletion and low-nadir CD4^+^ lymphocyte count (common deletion^+^ 145 ± 169, common deletion^−^ 250 ± 186, *P* = 0.07) and tubular dysfunction (study participants with both phosphate and urate wasting; common deletion^+^ 5/11, 45%; common deletion^−^ 2/21, 10%; *P* = 0.07). Among TDF-treated study participants there was no effect of pharmacokinetic boosting agents on the presence of the common deletion. No other large-scale mtDNA deletions were detectable by long-range PCR.

## Discussion

We have studied mtDNA markers in the urine of HIV-infected study participants, considering both mtDNA content and mtDNA mutations. First, we have demonstrated that the measurement of mtDNA from urine is feasible in the HIV clinic setting. Although mtDNA depletion has historically been considered the hallmark of drug-induced mitochondrial dysfunction, this did not prove a useful marker in this context. This was because the predominant driver of mtDNA content appeared to be differences in the cellular composition of urine samples. An analogous example has been documented in studies of peripheral blood mtDNA content in sepsis, where changes in mtDNA content are heavily influenced by changes in proportions of different leucocyte populations [17]. Of course, the putative cells of interest for our study, proximal tubular renal epithelial cells, are likely to only constitute a minority of the total cellular population of urine. Ideally, mtDNA would be examined in each cell population within urine; however, currently reliable methods do not exist for fluorescence-activated cell sorting of proximal tubule cells from a random urine sample.

In contrast, we saw a much clearer signal for the mtDNA common deletion mutation. This assay will not be significantly affected by changes in the cellular composition of urine, and can detect mitochondrial damage which only affects a small subpopulation of cells within the sample. We saw a clear relationship between the presence of the common deletion and TDF exposure. This is the first study to show such an association between TDF and mtDNA mutations. The assumption is that this signal is coming from tubular epithelial cells. As described above, a cell-specific approach such as fluorescence-activated cell sorting would be required to confirm this hypothesis. Furthermore, such an approach would allow us to exclude mtDNA proliferation as a possible association of deletions. MtDNA deletions are found at their highest levels in postmitotic tissues such as muscle and neurons as expansion of deletions occurs within these cells over time [18]. Within our study, the association between increased duration of TDF exposure and the common deletion is in keeping with this notion of accumulating mutations over time. Once deletions have formed they tend to persist, even after the causal factor (including NRTI therapy) has been removed [9]. It will be important for future studies to address whether these renal mutations persist after switch from TDF.

Our study has a number of limitations. Given that the common deletion was seen predominantly in TDF-treated study participants, we were limited in our power to detect the effects of other possible factors within this subgroup. For example, within our data there were borderline significant associations between the presence of the common deletion and tubular dysfunction, and between common deletion and nadir CD4^+^ lymphocyte count. The analyses of phosphate biochemistry are potentially limited by our use of random (unfasted) samples. This was a pragmatic decision as the primary aim of this study was to provide a proof-of-concept that mtDNA abnormalities could be detected in routine clinical samples.

In conclusion, our data demonstrate that mtDNA injury can be detected in the urine of TDF-treated patients. The degree to which this may drive TDF-associated renal pathology merits further investigation. The assessment of mtDNA mutations, such as the common deletion, in urine is simple and noninvasive, and potentially scalable to clinical practice. Further longitudinal studies should be performed to determine whether this marker of mitochondrial damage defines a subgroup of TDF-treated patients who are at risk of clinical renal complications. Given the recent availability of tenofovir alafenamide such investigations might in the future help to prioritize patients for treatment switch.

## Acknowledgements

This study was funded by the British Infection Association. B.A.I.P is funded by the Wellcome Trust (109975/Z/15/Z).

B.A.I.P., D.A.P., and J.A.S. designed the study. R.S., C.R.B., and B.A.I.P. conducted the study and analysed the data. B.A.I.P. wrote the manuscript which was revised and agreed by all authors.

### Conflicts of interest

There are no conflicts of interest.

## Supplementary Material

Supplemental Digital Content

## Figures and Tables

**Table 1:** Study participants characteristics.

	TDF ART (all, 24)	Non-TDF ART (11)	ART Naive (13)	TDF^+^CD^+^ (13)	TDF^+^CD^−^ (9)
Age (years)	45.3 ± 10.3	46.6 ± 14.3	36.5 ± 11.7	47.5 ± 10.1	45.1 ± 9.7
Male	16 (67%)	7 (64%)	12 (92%)	10 (77%)	5 (56%)
Time since HIV diagnosis (months)	149 ± 72	204 ± 110	18 ± 23	141 ± 39	175 ± 104
Time on treatment (months)	117 ± 53	161 ± 96		125 ± 38	123 ± 62
Time on TDF (months)	49 ± 32			63 ± 29	32 ± 29
Current CD4^+^ cell count (cells/μl)	637 ± 337	570 ± 237	464 ± 251	608 ± 296	729 ± 400
HIV-1 RNA viral load (<50 c/ml)	23 (96%)	8 (73%)		13 (100%)	8 (89%)
Nadir CD4^+^ cell count (cells/μl)	135 ± 98	172 ± 112	410 ± 230	116 ± 86	168 ± 120
Black African ethnicity	8 (33%)	3 (27%)	0	2 (15%)	5 (56%)
Current smokers	7 (29%)	3 (27%)	5 (39%)	4 (36%)	2 (25%)
Documented osteoporosis	0	1 (9%)	0	0	0
Hepatitis C coinfection	2 (8%)	1 (9%)	0	1 (8%)	1 (11%)
Type 2 diabetes mellitus	2 (8%)	2 (18%)	1 (8%)	1 (8%)	1 (11%)
Hypertension	3 (13%)	2 (18%)	0	1 (8%)	1 (11%)
eGFR (CKD-EPI)	93 ± 21	93 ± 21	101 ± 18	91 ± 17	91 ± 24
TmP/GFR (mmol/l)	0.82 ± 0.28	1.03 ± 0.30	1.02 ± 0.23	0.86 ± 0.29	0.75 ± 0.31
FEUa (%)	9.1 ± 3.3	8.3 ± 5.3	8.7 ± 6.6	9.2 ± 2.5	8.3 ± 4.4
UPCR (mg/mmol)^a^	11.5 ± 4.1	13.8 ± 8.4	14.9 ± 11.2	11.4 ± 5.0	12.2 ± 2.2
RBP (mg/l)	0.6 ± 2.1	0	0	0.3 ± 1.1	1.1 ± 3.2

Where shown, values are mean ± SD. In two TDF-treated study participants molecular data was not able to be included. ART, antiretroviral therapy; TDF, tenofovir disoproxil fumarate; CD^+^/CD^−^, mtDNA ‘common deletion’ detected/not detected; eGFR, estimated glomerular filtration rate; TmP, tubular maximum resorption of phosphate; FEUa, fractional excretion of urate; UPCR, urinary protein:creatine ratio; RBP, urinary retinol binding protein.

^a^One TDF-treated case with nontubular proteinuria was excluded.
