# Supplementary material for: Tenofovir disoproxil fumarate-associated renal tubular dysfunction: noninvasive assessment of mitochondrial injury
Source: AIDS. 2017 May 11;31(9):1297–301. doi: 10.1097/QAD.0000000000001466 (PMC5427982; doi:10.1097/QAD.0000000000001466)
Supplement: Supplemental Digital Content [file aids-31-1297-s001.docx]

**TDF associated renal tubular dysfunction: non-invasive assessment of mitochondrial injury**

Samuels *et al.*

*Supplementary Material*

*Mitochondrial DNA content in urine.*

Urine is a highly heterogeneous tissue which may vary widely in cellular composition between individuals. It is likely that the cell type of interest (proximal tubular (PT) cells) is only a minority population within a urine sample. In keeping with this notion, we observed no difference in urine mtDNA depletion between ART groups (log_10_ mtDNA copies/cell ±SD; TDF+, 2.75 ±0.73; TDF-, 2.61 ±0.81; p=0.6), or according to renal biochemistry.

In order to more fully explore the relationship between urinary cellular content and mtDNA content we then examined the correlation between these variables. This analysis revealed a complex relationship. Cellular content in urine ranged from <0.77 to 4.48 log_10_ cells/mL. Cellular content did not differ according to TDF exposure. As expected, cellular content and total mtDNA content per mL of urine were positively correlated (r 0.52, p=0.001). However, we saw a strong inverse correlation between mtDNA content per cell and the cellular content of urine (r -0.73, p<0.001, **Supplementary Figure**).
